# The Burden of COVID-19 Mortality Due to Referrals From Skilled Nursing Facilities in a Small Community Hospital

**DOI:** 10.7759/cureus.42369

**Published:** 2023-07-24

**Authors:** Amir R Reihani, Jacob Vanhouten, Angad S Gill, Kanwar Arora, Edward Manning

**Affiliations:** 1 Pulmonary Diseases, Yale-Affiliated Griffin Hospital, Shelton, USA; 2 Pulmonary and Critical Care Medicine, Eisenhower Hospital/University of Riverside, Rancho Mirage, USA; 3 Public Health, Griffin Hospital/Yale University, New Haven, USA; 4 Internal Medicine, Griffin Hospital, Derby, USA; 5 Pulmonary and Critical Care Medicine, Yale New Haven Hospital, New Haven, USA

**Keywords:** covid-19, adverse outcomes, community hospital, skilled nursing facility, morbidity and mortality

## Abstract

Background: Amidst the COVID-19 pandemic, nursing home residents have seen a significant increase in hospitalizations. However, there is a lack of published data on the healthcare provided to these individuals in community hospitals. This knowledge gap hinders our understanding and evaluation of the quality and outcomes of care received by nursing home residents when they are hospitalized for COVID-19 or other medical conditions.

Furthermore, insufficient data is used to compare the clinical outcomes of COVID-19-related admissions from nursing facilities between small community hospitals and tertiary care facilities. It is essential to conduct further research to identify potential disparities, which may indicate an unequal burden of nursing facility referrals to less-resourced hospitals.

Objective: We examined the characteristics of COVID-19-related deaths in a community hospital during the first surge of COVID-19 and calculated the proportion of patients who expired and were transferred from nearby nursing facilities.

Method: We performed a retrospective review of all cases of COVID-19 admitted to a 160-bed community hospital in Connecticut from January 1, 2020, to August 1, 2020. One hundred seventy-seven patients with COVID-19 who were admitted to our hospital were included in this study. Seventy patients (70/177, 39.54%) were transferred from nearby nursing facilities. The primary objective of this study was to examine the clinical characteristics of COVID-19-related deaths in our community hospital during the first surge of COVID-19. We also calculated the proportion of patients who expired and were transferred from nearby nursing facilities.

Results: Although the mortality rate in our community hospital was 15.23% (27/177), the majority of those who died were from nursing facilities (85.18%, 23/27). In contrast, mortality among the patients admitted from the community was 3.7% (4/107). The patients transferred from a nursing facility had 12.6 times higher odds of 30-day inpatient mortality or referral to hospice (95% CI, 4.1-38.5; p<0.001).

Conclusion: The majority of COVID-19 deaths in our community hospital were due to nursing facility referrals. We hypothesize that this high mortality may reflect healthcare inequality due to the unequal burden of nursing facility referrals to less-resourced hospitals.

## Introduction

By August 2020, there were 4.4 million cases of SARS-CoV-2, the virus that causes COVID-19, in the United States resulting in more than 151,265 [1] deaths. Connecticut was among the states most affected by the early pandemic. The literature [2-4] shows that older adults with multiple comorbidities were particularly vulnerable to COVID-19. Skilled nursing facilities (SNF) and long-term care (LTC) facilities [4,5] are home to members of this vulnerable population. While many northeastern states had severe outbreaks in LTC facilities, Connecticut had the highest [6] reported death rate with 91 per 100,000 total population. Cumulative data presented to the Connecticut Department of Public Health (DPH) on August 4, 2020, reported that 8,788 nursing home residents in Connecticut tested positive for COVID-19, of which 2,855 died, a mortality rate of 32.5%, which is considerably higher than the general population with 1.8% [6,7] mortality. This may highlight the underpreparedness [8] to face such emergency situations among SNF and LTC facilities. While the rate of hospitalization of nursing home residents increased during COVID-19 [9,10], there is a paucity of literature [11-14] examining its impact on community hospitals. Anecdotally, we felt that there was a large proportion of COVID-19-related admissions and deaths at our community hospital from nursing homes. Therefore, we examined the clinical characteristics of COVID-19-related deaths in a community hospital during the first surge of COVID-19 (until August 2020) and calculated the proportion of patients who expired and were transferred from nearby nursing facilities.

## Materials and methods

We conducted a comprehensive retrospective review of all COVID-19 cases admitted to a 160-bed community hospital between January 1, 2020, and August 1, 2020. Prior to commencing the study, we obtained approval from the institutional review board (IRB) to ensure adherence to ethical guidelines.

To confirm the diagnosis of COVID-19, we employed the polymerase chain reaction (PCR) method, which is widely recognized as the gold standard for detecting the SARS-CoV-2 virus. This diagnostic technique provides high sensitivity and specificity in identifying COVID-19 cases.

For our analysis, we collected both clinical and laboratory information related to the patients. This comprehensive dataset allowed us to perform a thorough examination of various factors and their associations with COVID-19 outcomes. The data collected included demographic information, medical history, symptomatology, laboratory test results, and treatment interventions.

Descriptive statistics were employed to summarize the collected data and present the results in a meaningful manner. Continuous variables, such as age and laboratory measurements, were reported as medians with interquartile ranges (IQRs) to account for potential outliers and provide a robust representation of the data. Categorical variables, on the other hand, were summarized using counts and percentages.

To explore the associations between specific variables, we calculated odds ratios. This statistical measure allowed us to evaluate the strength and direction of the relationships between different factors and COVID-19 outcomes. To determine the statistical significance of these associations, we utilized Fisher's exact test. A p-value threshold of less than 0.05 was considered as the criterion for statistical significance.

The data analysis for this study was performed using Statistical Package for Social Sciences (SPSS) version 22.0 (IBM SPSS Statistics, Armonk, NY), a widely utilized statistical package known for its comprehensive capabilities in analyzing complex datasets. We employed this software to conduct the necessary statistical tests, perform data cleaning and transformation, and generate relevant graphical representations. By employing rigorous data collection methods, robust statistical analyses, and the use of established software, we aimed to ensure the reliability and validity of our findings. The comprehensive nature of our study and the meticulous approach taken in data analysis contribute to the strength and credibility of the results obtained.

## Results

One hundred seventy-seven patients with COVID-19 were admitted to our community hospital from January 1, 2020, to August 1, 2020. All eligible patient charts were reviewed. Seventy patients (39.54%) were transferred from nearby nursing facilities. Although the mortality rate in our community hospital was only 15.23% (27/177), a significant majority of the deceased were those who were transferred from nursing facilities (85.18%, 23/27). In contrast, the mortality rate among the patients who were admitted from the community was only 3.7% (4/107) (Table 1). The patients transferred from a nursing facility had 12.4 times higher odds of 30-day inpatient mortality or referral to hospice (95% CI, 4.1-38.5; p<0.001).

**Table 1:** Baseline characteristics of the patients with COVID-19 infection who expired in a small community hospital in southwestern Connecticut, United States, during the first surge of COVID-19 (January 1, 2020, to August 1, 2020)* *Data presented as median (interquartile range) or number (percentage) SNF, skilled nursing facility; LTC, long-term care

Total COVID-19 admissions during the first surge	177
Mortality among all COVID-19 admissions	27 (15%)
Total number of COVID-19 admissions that were referred from SNF or LTC facilities	70 (40%)
Number of deaths among SNF or LTC facility referrals compared to the total number of deaths	23/27 (85%)

The majority of the deceased had a body mass index (BMI) of >25 (77%). Hypertension was the second most common comorbid condition (71.42%), followed by diabetes (46.4%). Congestive heart failure was also a common comorbidity (35.7%). Twenty-three patients among the deceased developed acute respiratory distress syndrome (82.1%). The code status of the majority of the deceased was switched to comfort measures prior to death (24/27, 88.8%). Among all admitted patients who were transferred from a nursing facility, 31% died (23/70). The majority of the patients who were referred from SNF that died presented with oxygen saturation of less than 94% (24/27, 89%) and altered mental status (24/27, 89%) upon presentation to the emergency department. We also compared mortality rates among the patients who were referred from nursing facilities based on demographics and comorbidities (Table 2). Among the 70 patients who were transferred from a nearby nursing facility, 23 died (23/70, 32%). The median age of death among the referral from nursing facilities, regardless of outcome, was 80 years old. Heart failure and diabetes were slightly more common among the deceased group, compared to those who survived (Table 2).

**Table 2:** Demographics and major comorbidities among the study participants *Data presented as median (interquartile range) or number (percentage)

Demographics and major comorbidities of nursing facility residents admitted to a small community hospital in southwestern Connecticut, United States, during the first surge of COVID-19 (January 1, 2020, to August 1, 2020)*	Outcome	
	Survived	Died
	47 (67%)	23 (32%)
Age	80 (76-85)	80 (72-88)
Body mass index	27.75 (23.43-31.28)	26.5 (23.95-27.70)
Length of stay	10 (6-14)	8 (5-12.5)
Major comorbidities		
Hypertension	32 (84%)	19 (83%)
Congestive heart failure	11 (29%)	8 (35%)
Diabetes mellitus	15 (39%)	10 (43%)
Body mass index of >30	15 (39%)	4 (17%)
Chronic obstructive pulmonary disease	8 (21%)	4 (17%)

## Discussion

We studied the clinical characteristics of COVID-19-related deaths among referrals from SNF and LTC facilities to a community hospital during the first surge of COVID-19 (January 1, 2020, to August 1, 2020). We evaluated the clinical characteristics of all patients who died from COVID-19. Our study revealed that the vast majority of patients who died were referred from SNF (23/27, 85%). We also analyzed the clinical characteristics of nursing facility referrals and outcomes. The high proportion of nursing facility referrals to our community hospital during the first surge of COVID-19 in 2020 highlights the crucial role of community hospitals in healthcare delivery. Our 160-bed university-affiliated community hospital is located in Derby, Connecticut. This acute care community hospital serves more than 130,000 residents of the Lower Naugatuck Valley Region, which straddles parts of Fairfield, New Haven, and Litchfield County. These regions were also among the highest rates of hospitalization [7] and associated death during the 2020 pandemic.

In early pandemics, a lack of emergency preparedness in Connecticut nursing homes, like many states, was evident in multiple aspects, including a shortage of staff, equipment, and care protocols. Consequently, there was an influx [6] of nursing facility referrals to nearby hospitals. However, national data regarding 30-day mortality among patients transferred from a nursing facility is limited. In a recent cohort study, Asch et al. [15] assessed the clinical characteristics of mortality among 38,517 adults with COVID-19 who were admitted to 955 US hospitals from January 1, 2020, to June 30, 2020. They evaluated 892 urban and 63 nonurban hospitals all over the United States. The results of that study showed that the patients transferred from a nursing facility had 2.43 times higher odds of 30-day inpatient mortality or referral to hospice than those admitted from the community (95% CI, 2.22-2.65; p<0.001). However, our study in this community hospital showed that the patients transferred from a nursing facility had 12.4 times higher odds of 30-day inpatient mortality or referral to hospice (95% CI, 3.9-52.2; p<0.001 {Fisher's exact test}). Moreover, Asch et al. [15] reported that outcomes for patients with COVID-19 rely not only on individual-level risk factors but also on the hospital where care is received. They did not categorically compare [15] the clinical characteristics of alive discharged patients who died to all individuals who were transferred from nursing facilities.

In general, due to differences in public hospitals' resources and infrastructure, the clinical characteristics of COVID-19-related hospitalization in small community hospitals may differ from their larger urban or academic counterparts. There is a scarcity of data in the literature that compares explicitly the clinical outcomes of COVID-19-related admissions among nursing facility referrals to acute care hospitals. Healthcare delivery disparities between states, urban communities, and socioeconomic status are well known, including care received at community care hospitals [16] versus larger tertiary care facilities. We hypothesize that the COVID-19 pandemic exacerbated these disparities, resulting in poorer outcomes for nursing facility residents suffering from COVID-19. Our next step is to identify the proportion of nursing home COVID-19-related referrals to nearby hospitals in our area to compare the rates of COVID-19-related admissions between community care and tertiary care facilities and outcomes.

This study has several limitations that require careful consideration. Fundamentally, it is essential to note that this study was conducted during the early stages of the pandemic in a community hospital, where there was no national consensus regarding treatment guidelines and laboratory parameters. Consequently, not all laboratory data, including key markers such as interleukin 6 and procalcitonin, was collected. The retrospective design of the study further restricted the availability of complete laboratory tests for every patient. As a result, certain variables such as C-reactive protein (CRP), erythrocyte sedimentation rate (ESR), procalcitonin, and interleukin 6 had to be removed from the analysis due to a significant number of missing values. Although some of these variables have been previously reported, their exclusion from the analysis was necessary due to data limitations.

Additionally, it is important to acknowledge that this study was exclusively conducted at a small community hospital in the northeastern United States. The hospital's close proximity to a nearby nursing home may have led to a higher referral rate of sicker patients to this particular center. Unfortunately, precise data regarding the percentage of referrals to the community hospital compared to tertiary counterparts could not be obtained. Consequently, there is a potential introduction of selection bias, which restricts the generalizability of the findings to a broader population.

Furthermore, it is worth noting that a substantial proportion of patients included in this study presented with severe disease upon admission, primarily due to advanced age and multiple comorbidities, including advanced dementia in some cases. This circumstance hinders our models' ability to accurately assess the relationship between independent variables and mortality when compared to the patients with milder disease. Moreover, the applicability of our results to populations with a higher baseline functional status is limited.

## Conclusions

The majority of COVID-19-related deaths at our community hospital during the first surge of COVID-19 in our area, from January 1, 2020, to August 1, 2020, were due to admissions from nursing homes. Nursing home referrals during the COVID-19 pandemic have placed a heavy burden upon the resources of our hospital and likely other similar hospitals. The lack of published data on COVID-19-related admissions from nursing facilities to acute care hospitals makes it difficult to investigate potential health disparities. A better understanding of the clinical characteristics of COVID-19-related mortality in small community hospitals and larger tertiary care facilities within the same geographical area will assist in the regional planning of acute care referrals from nursing facilities and preparing additional strategies to reduce adverse outcomes. It will also allow community hospitals to be better prepared to reduce adverse outcomes. In conclusion, we aim to foster the expansion of scientific inquiry through the publication of this report, urging fellow researchers to contribute to our understanding of healthcare disparities among the patients referred from nursing facilities, with a specific focus on COVID-19-related admissions.
